# "Semicut” skill on the cystic duct in laparoscopic cholecystectomy

**DOI:** 10.3389/fsurg.2022.1004290

**Published:** 2023-01-06

**Authors:** Linxun Liu, Zhanxue Zhao, Jinyu Yang

**Affiliations:** Department of General Surgery, Qinghai Provincial People's Hospital, Xining, China

**Keywords:** laparoscopic cholecystectomy, residual tones in cystic duct, "semicut" skil, gallstone, cystic duct stone

## Abstract

This study aimed to decrease the incidence of residual stones in the cystic duct and consequently decrease the incidences of intractable pain and the formation of a small gallbladder after laparoscopic cholecystectomy (LC). We changed the order of the clamps when performing LC, used the “semicut” skill of the cystic duct, and removed the stones residing in the cystic duct. A total of 45 patients underwent the operation, and all operations were completed successfully. This technique did not increase the operation time or difficulty. In conclusion, the “semicut” skill of the cystic duct is a safe and feasible surgical method that may change the occurrence of intractable pain after LC.

## Introduction

Cholecystectomy is the main surgical method for the treatment of cholecystolithiasis. Unfortunately, between 5% and 47% of patients present with recurrent gastrointestinal symptoms post-operatively, such as abdominal pain, dyspepsia, nausea, vomiting, fever, diarrhea, and jaundice (1). Residual gallstone in cystic duct is one of the causes of the above symptoms. The spiral valvular structure in the cystic duct protects cholecystoliths from falling into the common bile duct and makes the small stones remain in the cystic duct, resulting in residual stones in the cystic duct, intractable pain, and the formation of a small gallbladder after laparoscopic cholecystectomy (LC) (2). The literature has reported a high incidence of residual gallstones in cystic ducts after laparoscopic cholecystectomy of approximately 12.3% (3). However, this was reported 21 years ago, and there is no detailed statistics at present. When LC is widely utilized, the incidence of residual gallstones in the cystic duct will gradually increase (4). Surgeons should have a strong awareness of the location of the gallstones within the cystic duct (5). We changed the clamp action and the sequence of cystic duct clamping during LC, which we call the “semicut” skill of the cystic duct. This study aimed to introduce a novel and safe method for assessing the anatomy of the gallbladder triangle. We hope to accumulate data and assess whether there will be a reduction in intractable pain in patients in the future.

## Materials and methods

### Patients

This study included 45 patients (men, 17; women, 28; age, 27–76 years) with an average age of 51 years who presented in the general surgery department of Qinghai Province People's Hospital from June 2021 to February 2022. All patients had a history of gallstones for 3–15 years. Before the operation, 31 patients underwent routine abdominal color Doppler ultrasonography, which showed the presence of single or multiple gallstones, the largest of which was 33 mm, without intrahepatic bile duct or common bile duct stones, and there was no common bile duct coarsening in the patients; meanwhile, gallstones in the gallbladder duct were observed in two patients. Twelve patients with gallstone pancreatitis were admitted to our department. Seven patients were diagnosed with gallbladder enlargement with gallstones and acute pancreatitis by abdominal color Doppler ultrasonography and abdominal CT examination. The other five patients were diagnosed with gallbladder enlargement, acute pancreatitis, and peripancreatic exudation by abdominal CT examinations. All patients underwent elective LC.

### Operative method

First, patients were treated by undergoing general anesthesia with endotracheal intubation, and they were positioned with their heads kept high, and their feet placed low. The conventional three-hole method was used, and the artificial pneumoperitoneum pressure was set at 12–14 mmHg. In patients with high tension in the gallbladder, puncture and decompression were performed first, and adhesions around the gallbladder and the dissected gallbladder triangle were separated. We set the junction of the gallbladder neck and the cystic duct as the distal end of the cystic duct, whereas the junction of the cystic duct into the common hepatic duct was set as the proximal beginning of the cystic duct. Stones at the gallbladder neck have a great impact on the triangular dissection of the gallbladder. The gallbladder or the gallbladder ampulla was cut to remove the stone first, and the gallbladder duct was dissected to the distal part of the gallbladder duct. The distal part of the gallbladder duct was gently pinched and handled with the separating forceps. We summarized the steps in the treatment of a cystic duct during LC operation as follows: (1) When handling the cystic duct containing stones, the stones should be pushed and squeezed gently into the gallbladder, and then the titanium clip should be clamped at the distal end of the cystic duct. (2) If the stone is incarcerated and cannot be pushed out of the cystic duct, the cystic duct should not be pinched excessively, or the stone fragments will drop into the common duct unless the titanium clip is clamped at the distal end of the cystic duct. (3) If the stones are embedded at the proximal beginning of the cystic duct, the titanium clip should be clipped at the distal end of the cystic duct to prevent the gallbladder stones from falling into the cystic duct again, and the titanium clip should be as close as possible, leaving a long enough cystic duct for any later operations. (4) If there are no stones in the cystic duct, the titanium clip should be clamped at the distal end to prevent the gallbladder stones from falling into the cystic duct, and the titanium clip should be as close as possible (1), (3), (4). To complete the operation, the following operations should be performed: The anterior wall of cystic duct should be cut in half to make sure the incision is far away from common bile duct; that is, the cystic duct “semicut” skill. The stones should be extruded with separating forceps from the incision or the small stones may be milked out of the cystic duct by the natural pressure of the biliary bile and smooth and clear flow of bile should then be observed. After that, a titanium clip should be clamped between the incision and approximately 0.5 cm from the common bile duct. Finally, the gallbladder should be removed from the gallbladder bed routinely, and a negative pressure drainage tube should be placed in the abdominal cavity.

## Results

The operation lasted 30–60 min, with an average of 45 min. Two patients diagnosed with cystic duct stones by color Doppler ultrasonography had their stones successfully removed by this method. Seventeen of the remaining 43 patients had suspected cystic duct stones because the bile removed from the incision was not clear during the operation. Using this method, 10 patients were confirmed to have cholelithiasis, and the stones were successfully removed. The remaining 26 patients did not have any cystic duct stones that were found during the operation but still underwent the “semicut” technique. We found white flocculent bile with sand flowing out of the cut cystic duct in these patients. Although it is impossible to judge whether the cystic duct contains stones according to the principle of gallstone formation, we believe that they will eventually develop into stones in the residual cystic duct in the future. All patients left the hospital within 3–5 days after the operation. None of the patients developed abdominal pain or jaundice of the skin related to stones or biliary tract infections during hospitalization. After the operation, routine blood biochemical examinations were performed, including total, direct, and indirect bilirubin levels. Among the patients, four had an elevated total bilirubin of up to 79.6 µmol/l, accompanied by mildly elevated alanine aminotransferase and glutamic oxaloacetate aminotransferase. The direct bilirubin/total bilirubin level was less than 50%. The cause of jaundice was related to a surgical stress reaction or transient liver functional damage. The blood biochemistries returned to normal 2 days after symptomatic treatment. The drainage tubes were removed 3 days after the operation in the patients if there were no complications. Patients were interviewed by telephone, and no patient had a fever, jaundice of the skin, or right epigastric discomfort. A follow-up period from 3 to 12 months from the operation showed that none of the patients developed the above symptoms, and the patients had recovered well.

## Discussion

**Figure 1:**
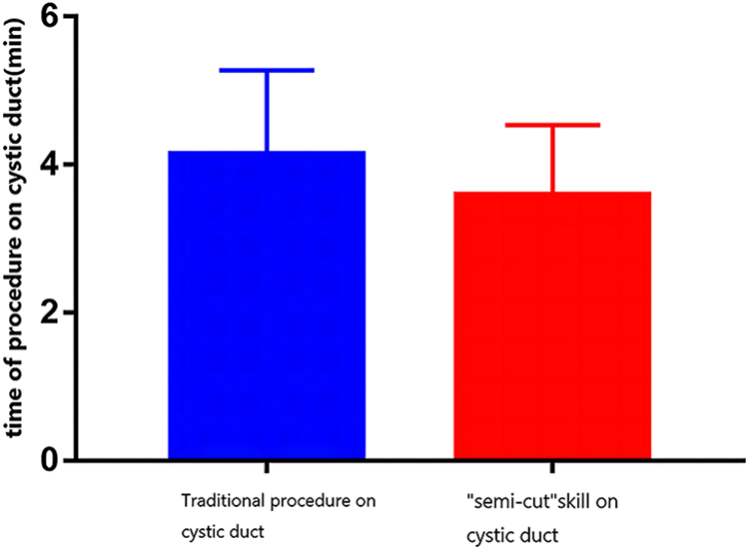
Comparison of the surgical time between the two procedures. No significant difference was found between the two groups.

**Figure 2:**
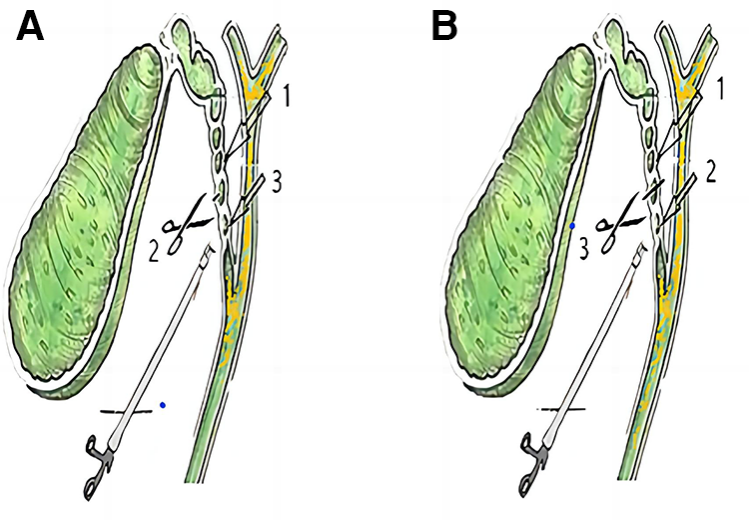
Order of the clamp placements on the cystic duct. (**A**) “semicut” skill on the cystic duct; (**B**) traditional procedure on the cystic duct.

The reasons for the occurrence of residual gallstones in the cystic duct include the instruments, the experience of surgeons, and their proficiency in performing LC operations. Patients often worry that LC cannot “remove the whole” gallbladder compared with open surgery. Some hospitals lack the conditions for intraoperative choledochoscopy, but large center hospitals also have problems regarding the size and location of cystic duct stones during LC operation because of the lack of inspection of the anatomical structure of the cystic duct (6). We found that 22% (10/45) of patients with cystic duct stones had their stones removed by the cystic duct “semicut” skill, and this incidence was close to the reported incidence of residual cystic duct stones. If LC had been performed routinely, these 10 patients would have had cystic duct stones that were likely to be left or partially left in the process, but now, we can remove them completely. If the stones are broken during pinching, the remaining stones will be “washed out” by the flow of the bile, which can effectively reduce the occurrence of residual stones in the cystic duct. The cystic duct “semicut” skill is a modification of the order and cutting method. After using this modification, the operation time was not prolonged. The cystic duct “semicut” skill only takes 3–5 min, averaging 4 min. There was no difference in the time of traditional LC compared to the new method (Figure 1). This new method can be quickly mastered by doctors who have mastered laparoscopic cholecystectomy, and this technique only changes the order of the placement of the clamps (Figure 2). Stones located in different parts of the cystic duct should be handled by different methods. The cystic duct “semicut” skill does not influence the operation, especially during the clamping of the titanium clip. Remarkably, the remaining wall of the cystic duct after cutting should not be too “thin” to avoid the disruption of the cystic duct, which could influence any subsequent operations. Therefore, we did not quantify the size of the “semicut” skill. For patients with thinner cystic ducts, we believe that this technique is not necessary; in these patients, only separation forceps are needed to squeeze the cystic duct so that even small stones are removed from the common bile duct because the stones have the same size as Oddis sphincteral stones. If the cystic duct is larger than 1.5 cm during laparoscopy, the cystic duct “semicut” skill can be used. After performing the cystic duct “semicut” skill, the bile duct is rarely disrupted, and the stones in the cystic duct can easily flow out from the incision; therefore, this method is suitable for use with LC.

In conclusion, the “semicut” skill of the cystic duct is a safe and feasible surgical method that may change the occurrence of intractable pain after LC. We will follow the current patients and reassess them in 5 years to determine whether this new technique can reduce the incidence of epigastric discomfort caused by stones in the cystic duct.

## Data Availability

The datasets presented in this study can be found in online repositories. The name of the repository is Dryad and accession number is doi:10.5061/dryad.9s4mw6mmd.
